# Development of Low-Molecular-Weight Gelator/Polymer Composite Materials Utilizing the Gelation and Swelling Process of Polymeric Materials

**DOI:** 10.3390/gels10050298

**Published:** 2024-04-26

**Authors:** Yutaka Ohsedo, Chinatsu Takagi

**Affiliations:** 1 Division of Engineering, Faculty of Engineering, Nara Women’s University, Kitauoyahigashi-machi, Nara 630-8506, Japan; 2 Faculty of Human Life and Environment, Nara Women’s University, Kitauoyahigashi-machi, Nara 630-8506, Japan

**Keywords:** fillers, gelation process, low-molecular-weight gelators, polymer composite materials

## Abstract

The creation of polymer composite materials by compositing fillers into polymer materials is an effective method of improving the properties of polymer materials, and the development of new fillers and their novel composite methods is expected to lead to the creation of new polymer composite materials. In this study, we develop a new filler material made of low-molecular-weight gelators by applying a gelation process that simultaneously performs the swelling (gelation) of crosslinked polymer materials and the self-assembly of low-molecular-weight gelators into low-dimensional crystals in organic solvents within polymer materials. The gelation process of crosslinking rubber-based polymers using alkylhydrazides/toluene as the low-molecular-weight gelator allowed us to composite self-assembled sheet-like crystals of alkylhydrazides as fillers in polymeric materials, as suggested by various microscopic observations, including infrared absorption measurements, small-angle X-ray diffraction measurements and thermal analysis, microscopy, and infrared absorption measurements. Furthermore, tensile tests of the composite materials demonstrated that the presence of fillers improved both the Young’s modulus and the tensile strength, as well as the elongation at yield. Additionally, heat treatment was shown to facilitate filler dispersion and enhance the mechanical properties. The findings demonstrate the potential of self-assembled sheet-like crystals of low-molecular-weight gelators as novel filler materials for polymers. The study’s composite method utilizing gelators via gelation proved effective.

## 1. Introduction

Low-molecular-weight gelators (LMWGs) are small molecules that can form molecular gels and gel-like substances by gelating water or solvents [1,2,3,4]. The physicochemical properties of gels, such as gelation behavior and gelation states, and their fundamental applications in various functional materials are being studied [5,6,7,8,9,10,11,12]. For example, the application of molecular hydrogels made of LMWGs containing π-electron conjugated aromatic systems in functional material chemistry, such as for photo- and electro-active functional materials, has been reported [13,14,15]. Further, molecular hydrogels comprising LMWGs containing fluorescence-emitting units and biocompatible units can be applied to sense biological substances [15,16]. Molecular hydrogels can be used as a base material for pharmaceutical ointments with spreadable properties [17,18,19]. Moreover, injectable molecular hydrogels that contain pharmaceuticals can be used in the healthcare and medical fields [20,21]. LMWGs that can form molecular oil gels with petroleum can be used as oil fence materials for disaster prevention after an oil leakage accident [22]. Thus, the potential applications of LMWGs have attracted attention from a wide range of fields, from electronic materials to materials for solving environmental problems, and active studies are being conducted. The mesh structure of LMWGs, composed of microfibers obtained by the self-assembly of the LMWGs, is the basis for the gelation of molecular gels [1,2,3,4]. Active research focuses on obtaining novel LMWGs and creating functional molecular gels with specific properties through the molecular design and synthesis of the molecular structures of LMWGs. However, there are many limitations to creating new LMWGs that form molecular gels and improving the function of the obtained molecular gels. The correlation between the molecular structure of LMWGs and the physical properties of molecular gels should be clarified, and an efficient method for obtaining the new molecular gels is required.

We have been guiding the acquisition of new LMWGs by the molecular design of new molecular structures and the revalidation of existing low-molecular-weight compounds as new LMWGs to efficiently obtain new molecular gels. We have observed that mixing these homologs effectively improves the physical properties of molecular gels [23]. One of the most active methods to improve the properties of molecular gels is to form a network structure of molecular gels by mixing two or more kinds of well-designed LMWGs and using their supramolecular interactions [24,25,26,27,28,29,30,31,32]. Contrarily, we have found that mixing the homologs of LMWGs, alkylhydrazides, with simple molecular structures containing alkyl and hydrogen-bonding groups of different chain lengths in the same molecule improves the mechanical properties of molecular organogels and hydrogels and induces thixotropic properties [23]. Further, we have found that by appropriately adjusting the mixing ratio of LMWGs, alkylhydrazides can reduce the diameter of the fibers constituting the gel mesh structure; we speculate that the resulting increase in mesh density improves the mechanical properties of the obtained molecular gels [23].

In the polymer field, hydrazide derivatives have been used as cross-linkers in paints and adhesives (e.g., adipic acid hydrazide) [33], tire additives [34], and foaming agents [35], but recently they are of interest as hydrogen bonding functional groups responsible for gel formation in LMWGs. For example, LMWGs with multiple aromatic hydrazides in the molecule [36,37,38,39,40], LMWGs that detect fluorine ions [41], LMWGs of hydrazide derivatives of 12-hydroxystearic acid [42], LMWGs that exhibit ultrasound-induced gel-forming ability [43], LMWG systems with sustained drug release [44], and LMWGs composed of simple alkyl hydrazides [45] have been reported and have played an important role in the functionalization of LMWGs.

This study examines a new method of using LMWGs as a filler material in polymers, i.e., a new composite method for filler composite polymer materials. Polymer composite materials have been extensively investigated as materials that combine the strength of inorganic materials and other polymer materials; they are easy to mold, are lightweight, and have characteristic mechanical properties [46]. Filler polymer composites comprise materials that are combined with polymer materials in the form of fillers. Fillers are inorganic or organic materials with particle diameters in the order of nanometers to micrometers, and when composited with polymer materials they can improve or modify various physical properties, including mechanical properties, of the underlying polymer material [47]. The most common fillers are naturally occurring clay minerals; silica; synthetic inorganic nanosheets; and carbon materials, such as carbon black and graphene. For polymer materials to be used stably under high loads over long periods, the physical properties should be improved and enhanced by compositing with fillers. However, fillers are used in the form of particles, sheets, and other low-dimensional materials, and their low dimensionality makes them susceptible to aggregation and agglomeration, which reduces the effectiveness of the fillers, and new methods of dispersing fillers in polymeric materials need to be considered [48,49].

In recent reports, carbon nanotubes, valued for their exceptional electronic properties [50], have emerged as a novel filler material. They can be combined with graphene to achieve radical scavenging in UV-protected waterborne polymer coatings [51], showcasing the successful integration of organic materials into functional fillers. Our ongoing research investigates the application of LMWGs, which have garnered attention in supramolecular chemistry, as fillers for polymeric materials [52,53]. We aim to explore LMWGs as potential new fillers and novel composite methods. Specifically, we examine whether the micrometer-scale fibers formed through LMWGs’ self-assembly can enhance the physical properties of polymer composites as fillers.

In this study, we compound polymers with LMWGs concurrently with the gelation of the LMWGs. Specifically, we apply a gelation process that simultaneously involves the gelation (swelling) of cross-linkable polymeric materials based on their swelling in organic solvents and gelation based on the self-assembly of fibers and the network formation of LMWGs in organic solvents. This process aims to create polymeric materials compounded with LMWGs. Hydrogel systems have been actively investigated in LMWG/polymer composite systems, mainly for biomaterial applications [52,53], and the swelling behavior of filler/rubber materials has been studied [54], but there has been no study of combining these with LMWGs, which form fillers by molecular self-assembly.

**Figure 1:**
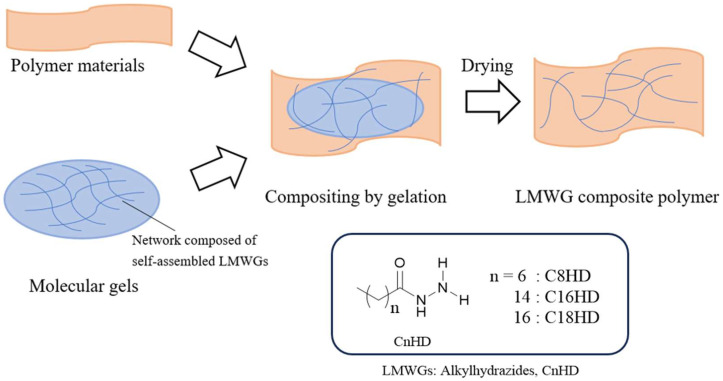
Gelation process for compositing fillers consisting of the self-assembled crystallization of low-molecular-weight gelators (LMWGs) into polymeric materials and the chemical structure of the LMWGs (alkylhydrazides).

To demonstrate the generality of this research concept, we selected crosslinked rubber-based polymeric materials for clothing, such as rubber bands and rubber fibers, as polymeric materials are swellable in organic solvents. As the LMWGs, we chose alkylhydrazides (C8HD, C16HD, and C18HD). Alkylhydrazides can gel toluene [45], a solvent capable of swelling rubber-based materials, and form micrometer-diameter and tens-of-micrometer-long crystalline structures as the structural elements of the gel, which can be expected to become fillers. We evaluated the mechanical properties of the obtained LMWG-compounded polymers through tensile tests and investigated the effectiveness of compounding, i.e., the filler effect of the LMWGs (Figure 1). Before this study’s LMWG/polymer system, a study applying a polymer gelator containing rotaxane moieties as fillers for polymer composite materials was reported [55].

## 2. Results and Discussion

Alkylhydrazides, which are LMWGs, were composited into operon rubber, known as rubber bands, and polyurethane fibers, which are polymeric materials. Alkylhydrazides gel is used in various organic solvents and forms good organogels in toluene [45]; crystalline materials in sheet or tape form with a few tens of micrometer diameter are the components of the gel networks in organogels [23]. As clay minerals composed of nanosheet crystals form a house-of-cards structure in aqueous solution to generate a gel state [56,57], it is speculated that low-dimensional crystalline materials composed of these LMWGs form a gel state in the same manner [23,45]. In this study, alkylhydrazides were selected as the LMWGs, and toluene was used as a solvent for composite preparation.

In the synthesis of this composite material, rubber polymers coexisted during the gelation process of toluene/LMWGs. Alkylhydrazide, rubber polymers, and toluene were combined in a screw-tube bottle, heated, then cooled to form toluene gels. Initially, heating dissolved alkylhydrazide crystals in toluene, swelling the rubber polymer. Upon cooling, the toluene solution transformed into a gel, enabling the formation of self-assembled crystals of alkylhydrazide within the polymer, serving as fillers. Previous research by the authors demonstrated toluene gel formation in alkylaldehyde single and mixed systems [23]. In this study, toluene gels formed in toluene solutions of alkylhydrazide in the presence of rubber polymers, indicating the introduction of self-assembled alkylhydrazide crystals into the polymer. The alkylhydrazide mixing ratio was C8HD/C16HD/C18HD:1/1/1 (*w*/*w*/*w*) for the tape-like component and C8HD/C16HD/C18HD:10/1/1/1 (*w*/*w*/*w*) for the fiber-like component, as previously examined, revealing differences in the gel network components [23,46]. The presence of alkylhydrazide was confirmed through sample weight changes and various microscopy techniques.

First, the compositing of alkylhydrazides into polymeric materials was confirmed by weight measurement, which showed an increase in the weight of the samples (Table 1). The weight of the rubber bands increased by 11–19 wt.%, and the operon rubber increased by 14–18 wt.%. These results suggest that the LMWGs could be included within the polymeric material. However, the increased weight was greater than that of the LMWGs incorporated in both polymeric materials (3 wt.%). This suggests that the LMWGs were concentrated within the hydrophobic polymeric material and that the high increase was due to the higher affinity of the polyurethane of the operon rubber for alkylhydrazides than for the rubber bands of the natural rubber raw material. Henceforth, samples will be denoted as RB for rubber bands and PU for operon rubber, and the composited LMWGs will be denoted as –CnHD... after the polymer materials RB or PU.

**Figure 2:**
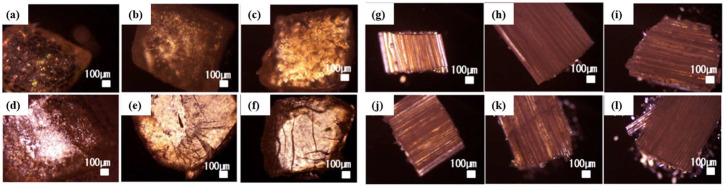
Polarized light microscopy of LMWG composite polymer samples under crossed Nicols: (**a**) RB, (**b**) RB-C8HD/C16HD/C18HD 10/1/1, (**c**) RB-C8HD/C16HD/C18HD 1/1/1, (**d**) RB-C8HD, (**e**) RB-C16HD, (**f**) RB-C18HD, (**g**) PU, (**h**) PU-C8HD/C16HD/C18HD 10/1/1, (**i**) PU-C8HD/C16HD/C18HD 1/1/1, (**j**) PU-C8HD, (**k**) PU-C16HD, (**l**) PU-C18HD.

Next, the compositing of the LMWGs into the polymeric material was confirmed using various microscopes. First, when the samples were observed under crossed Nicols by polarized light microscopy (Figure 2), more transmitted light was observed in the LMWG composite rubber band sample than in the LMWG-free rubber band sample. If a substance sandwiched between the crossed polarizer and analyzer (under crossed Nicols) allows light transmission, it indicates its orientational or crystalline state [58]. In such cases, as described below, X-ray diffraction measurements of the composite polymer revealed diffraction peaks corresponding to the molecular lengths of each alkylhydrazide, suggesting the presence of alkylaldehyde crystals, namely self-organized crystals of alkylhydrazides after the composite process, within the polymer. This confirmed the existence of an oriented structure other than the orientation structure of the polymer of the rubber band. This suggests that the polymer material of the LMWG composite rubber bands contains the self-assembled crystals of the LMWGs, such as those found inside the LMWG-derived molecular gels, and this oriented structure is responsible for the increase in transmitted light. However, in the LMWG composite operon rubber sample, the polymeric orientation structure of the operon rubber was strongly observed; further, no significant increase in transmitted light was observed by the LMWG composite, and the LMWG composite was not confirmed by polarized light microscopic observation.

**Figure 3:**
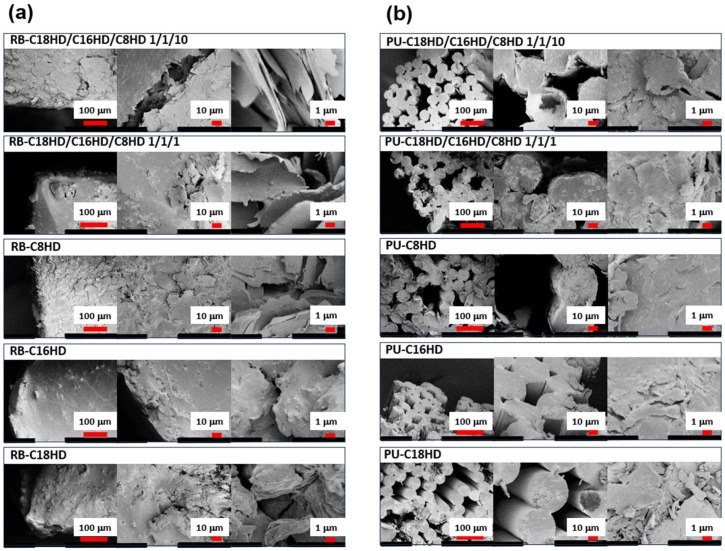
SEM images of the (**a**) RB and (**b**) PU samples.

Herein, we present scanning electron microscopy (SEM) observations of cross-sections of LMWG composite polymer samples, obtained by cutting with a cutter, to explore their internal microstructure (Figure 3). SEM images at the smallest scale on the right reveal a structure comprising overlapping sheets (with sub-micrometer thickness and lengths in the tens of micrometers) in the polymer material cross-section. Similar stacking of sheet-like structures at this scale has been observed in SEM images of xerogels obtained from alkylaldehyde gels [23,45]. These results suggest that the stacked sheets observed in the LMWG composite polymer cross-sections are self-assembled crystals of alkylaldehyde, consistent with subsequent X-ray diffraction measurements. Furthermore, in the rubber band system, the structures appeared finer in the mixed systems compared to the single systems, particularly evident in the samples using C8HD/C16HD/C18HD 10/1/1, as shown in Figure 3. Similar trends of refinement by changing the mixing ratio have also been observed in xerogel samples of alkylhydrazide-mixed toluene gels [23]. This trend of miniaturization by changing the mixing ratio was observed in the xerogel samples of the alkylhydrazide-mixed toluene gels. However, in this study, no tape-like or fiber-like crystals were observed, as in the xerogel samples of the alkylhydrazide toluene gels obtained from the alkylhydrazide mixed-system toluene solutions [23]. This indicates a difference in the self-assembly behavior of the LMWGs in solution systems and polymer systems. The formation of sheet-like crystal shapes within these polymeric materials may be because of the higher concentration (11–19 wt.%) compared with that (3 wt.%) in the solution system, which facilitated crystal growth from fiber- or tape-like to sheet-like shapes. These results indicate that alkylhydrazide gelators may form sheet-like self-assembled crystals within the polymer, which may form a house-of-cards structure-derived network structure within the polymer, similar to the gelation of alkylhydrazides [23,45].

**Figure 4:**
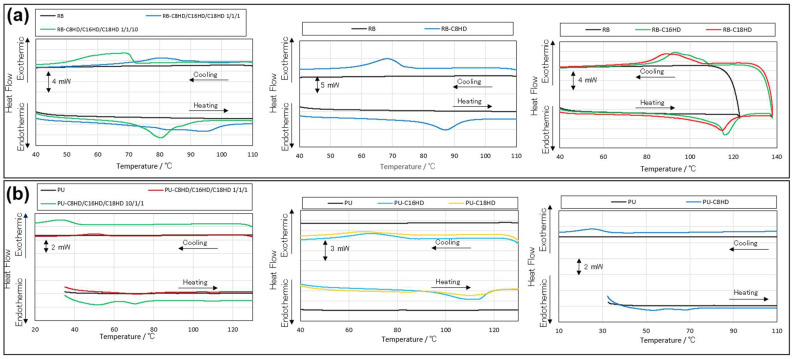
DSC curves of the (**a**) RB and (**b**) PU samples.

Further, the presence of the LMWGs within the polymeric materials was confirmed by thermal analysis, infrared absorption measurements, and small-angle X-ray scattering (SAXS) measurements. First, in the thermal analysis, no peaks based on heat in/out were observed in the differential scanning calorimetry (DSC) measurement results of the samples without the LMWGs composited in the polymeric materials (Figure 4 and Table 2). However, peaks based on exothermal activities during temperature increase and endothermal activities during temperature decrease were observed in the sample containing the LMWGs. Furthermore, the obtained peaks showed nearly identical ΔH values. Such results were also observed during the heating process of the alkylhydrazide toluene gels, involving dissolution into sol (dissolution of crystalline material into solution) and gel formation from sol during the cooling process (formation from solution of crystalline material) [23,45]. However, since the composite polymer samples were in a dry state, it is believed that the observed peaks correspond to the exothermic behavior during the melting of the self-organized crystals of the LMWGs formed within the composite polymer upon heating and the endothermic behavior during their formation upon cooling. This suggests that crystalline melting upon heating and crystallization upon cooling, similar to in the toluene solvent, occur within the polymer as well. Additionally, similar phenomena to those observed in toluene gels suggest the possibility of the formation of a network of crystals similar to gels during cooling in LMWG/polymer material systems. However, further investigation is required to determine whether there exists only a stacking of crystalline sheets or a network of crystallinity based on a house-of-cards structure.

**Figure 5:**
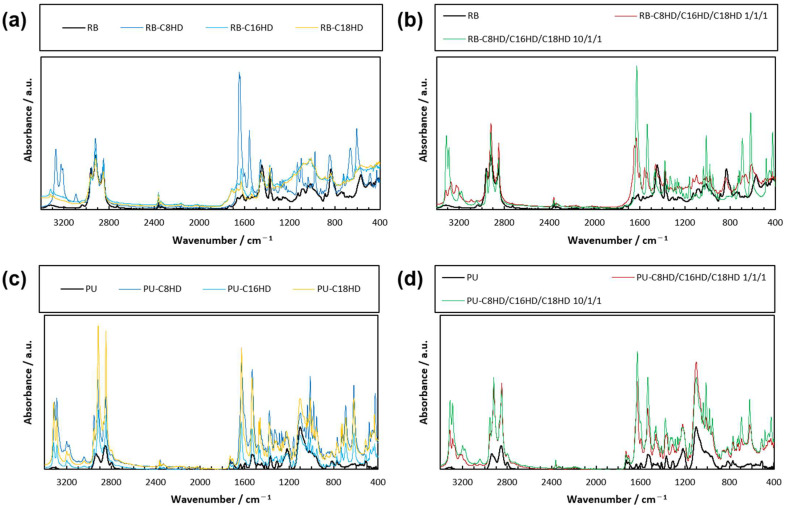
ATR–FTIR spectra of the (**a**,**b**) RB and (**c**,**d**) PU samples.

Infrared absorption measurements taken via attenuate total reflectance Fourier transform infrared spectroscopy (ATR–FTIR) showed that both LMWG composite samples exhibited many absorption bands that were not observed in the rubber-band-only or the operon-rubber-only samples (Figure 5). Characteristically, the LMWG composite samples showed absorption bands of stretching vibration corresponding to the CONH at 3200 cm^−1^ and the carbonyl group at 1600 cm^−1^, which are absorption bands [59,60] found in alkylhydrazides. This indicates that the polymer material contains alkylhydrazides. However, no difference was observed in the mixing ratio because the mixture comprised homologs. Additionally, no information on the formation of meshes between crystalline materials (information on intermolecular interactions) was observed.

**Figure 6:**
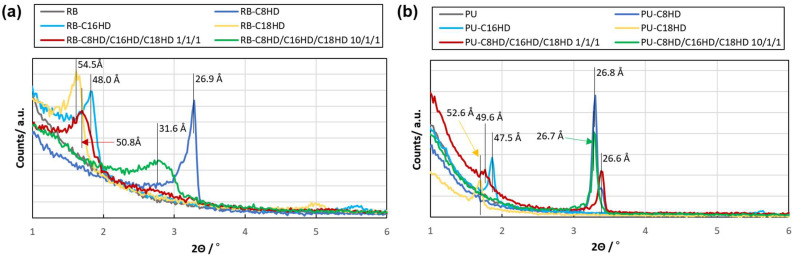
SAXS results of the (**a**) RB and (**b**) PU samples.

The results of the SAXS measurements showed diffraction peaks corresponding to C8HD: 27 Å, C16HD: 48 Å, and C18HD: 53–54 Å for the single LMWG composite samples and 1/1/1: 26–50 Å and 1/1/10: 27–32 Å for the mixture samples (Figure 6). These values are consistent with the literature values for the molecular length of alkylhydrazides [23,45]. In the single system, the length corresponded to two molecules in series, suggesting the existence of lamellar crystals in the LMWGs. However, in the mixed system, no peaks were observed, probably because of the interdigitated structure formed by the mixture of multiple components, and only diffraction peaks corresponding to the alkyl group structure were observed. These results are similar to those measured for toluene gels and xerogels of alkylhydrazides, suggesting the formation of crystalline materials within the polymeric materials.

**Figure 7:**
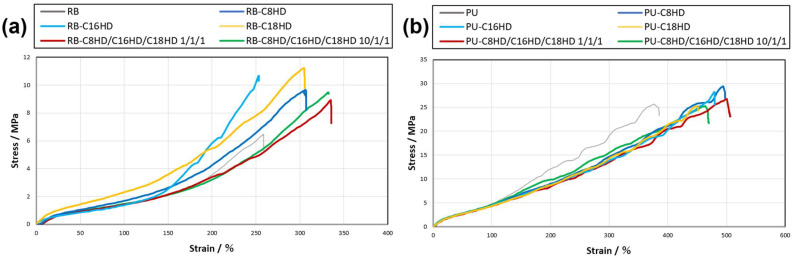
Tensile test results of (**a**) RB and (**b**) PU samples.

Next, mechanical property measurements, mainly tensile tests, were conducted on the LMWG composite samples to evaluate the LMWGs as a filler. When comparing rubber bands treated with LMWG composites and those treated only with toluene, the compositing process resulted in a comparable Young’s modulus for all samples except RB-C18HD while enhancing tensile strength by 1.4–1.7 times and elongation at yield by 1.2–1.8 times, except for RB-C16HD (Figure 7a and Table 3). This result suggests that the tensile strength of the rubber bands was improved by the LMWGs, i.e., by the filler formed by the LMWGs. Additionally, the maximum point stress was higher in the single system than in the alkylhydrazide mixed system. However, further investigation is needed to understand the causes behind the 1.8-fold increase in Young’s modulus for RB-C18HD and the similar elongation at yield for RB-C16HD.

In contrast, in the operon rubber sample (Figure 7b), the results for the operon rubber containing C8HD/C16HD/C18HD 10/1/1 were lower than those for the operon rubber with only toluene treatment. However, those with other low-molecular gelators were higher than those with only toluene immersion, i.e., 1.1 times higher than that of the toluene-only operon rubber. Additionally, the elongation at yield of the composite PU increased similarly to the composite rubber band system, reaching 1.2–1.3 times higher. Although the change was small compared with the 1.4- to 1.7-fold increase in tensile strength in the tests using rubber bands, the filler formed by the LMWGs improved the tensile strength of the operon rubber. No difference was observed between the single system and the mixed system or by mixing ratio, which may suggest that the compositing effect was not significant for the highly oriented fibrous rubber of the operon rubber.

The stress–strain curves of the tensile test results showed that the LMWG composite rubber bands had a higher strain than the rubber bands treated with only toluene. In particular, the amount of movement increased in the mixed system. The C16HD composite rubber bands had slightly less strain than the other samples; however, the slope of the graph was greater in the stand-alone system, suggesting that tensile strength was improved. Both stress and strain values were higher for the LMWG composite operon rubber than the LMWG-free toluene-treated operon rubber, indicating that the tensile strength of the LMWG composite operon rubber was improved. The graphs for the C8HD/C16HD/C18HD 1/1/1, C8HD, and C16HD composite samples had gradual slopes, which are inferred to be relatively soft. These results indicate that the filler effect [46,47] formed by the LMWGs improved the mechanical properties of the composite polymer samples.

**Figure 8:**
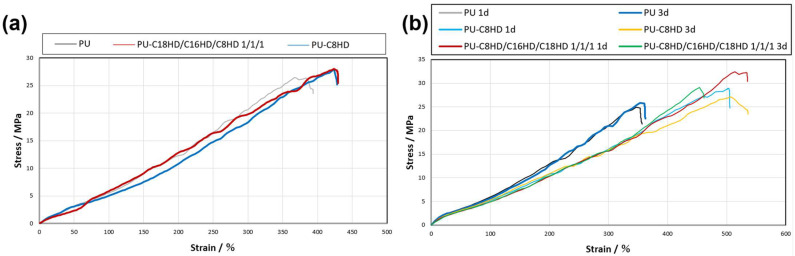
Tensile test results of (**a**) PU samples after repeated stretching operations and (**b**) PU samples after heat treatments (“d” represents day).

To verify the effect of the compounding process on the rubber properties, a tensile test was performed on a sample of the LMWG composite operon rubber after repeated stretching operations. The sample was stretched three times and returned to its original size (Figure 8a). Resultantly, the strain of the LMWG composite operon rubber sample was reduced by approximately 80%; however, there was no change in the maximum point stress compared with before stretching. These results indicate that the mechanical properties of the rubber material are mostly retained, although the rubber material becomes relatively stiff after the gelation treatment associated with the LMWG composite.

**Figure 9:**
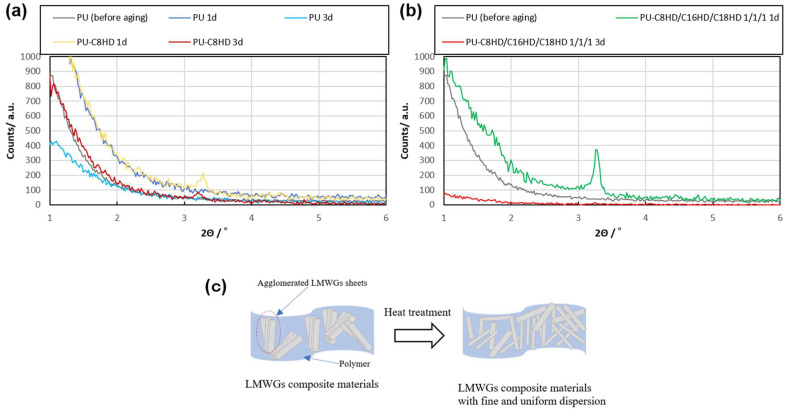
(**a**,**b**) SAXS results after heat treatment of PU samples; (**c**) schematic illustration of before and after the heat treatment of a PU sample.

Finally, we hypothesize that the sheet-like crystals originating from the LMWGs form a three-dimensional network structure within the operon rubber containing C8HD, C16HD, and C18HD using the method employed in this study, similar to gel formation in solution, due to the higher concentration of LMWGs compared to the gelation concentration. Thus, we attempted to homogeneously form the three-dimensional network structure of the sheet-like crystals in the polymeric material by reconstituting the network structure through heat treatment. The heat treatment temperature was set at 45 °C because the transition temperature of alkylhydrazides in the gel state during the temperature increase process was 40–50 °C. The heat treatment was performed for 24 and 72 h. As a result, although a decrease in Young’s modulus was observed after heat treatment, an improvement in tensile strength (1.1–1.3 times) and elongation at yield (1.2–1.4 times) was observed in all composite samples. Regarding the heat treatment duration, it was found that a decrease in tensile strength was observed in the 3-day samples, indicating that a duration of 1 day is sufficient. (Figure 8b, Table 4). The results of the SAXS measurements of the samples showed that the diffraction peaks were less visible than those before heat treatment, which was due to the fine and uniform dispersion of the sheet-like crystals by heat treatment (Figure 9a,b). These results suggest that aggregates of sheet-like crystals formed from LMWGs may loosen upon heat treatment, leading to uniform dispersion within the polymer material or crystal miniaturization, thereby potentially enhancing the mechanical properties of the composite samples as fillers (Figure 9c). However, the decrease in mechanical properties in the 3-day composite samples, juxtaposed with the disappearance of SAXS peaks, may indicate that in the 3-day samples the sheet-like crystal fillers have miniaturized to sizes below those that exhibit filler effects. Thus, the function of the LMWG filler in the polymeric material was enhanced by heat treatment.

## 3. Conclusions

In this study, polymer composite materials with self-assembled crystals of μm order diameter formed by LMWGs as fillers were obtained using a compositing method combining the organic solvent swelling of polymer materials and LMWG gelation inside polymers. Tensile tests showed enhanced mechanical properties in the LMWG composites, with alkylhydrazides forming sheet-like crystals in the polymers. Heat treatment further improved filler function by dispersing crystalline materials uniformly. These findings suggest the potential of the LMWG composite polymer material creation method via gelation for enhancing composite polymer material properties. While the filler effect of LMWGs in rubber-based materials was observed regarding mechanical properties, further investigation is needed to determine the effective size range, optimal shape, and concentration of fillers, as well as their impact on other material properties.

## 4. Materials and Methods

Alkylhydrazides, octanohydrazide (C8HD, 97%), palmitic acid hydrazide (C16HD, 98%), and stearic hydrazide (C18HD, 95%) were purchased from Tokyo Chemical Industry Co., Ltd., Tokyo, Japan, and were not further purified (numbers in parentheses indicate reagent purity). Toluene was purchased from Wako Pure Chemical Industries, Ltd., Tokyo, Japan, and was not further purified. The rubber ring band, “O’band, #16” (natural rubber, KYOWA LIMITED, Osaka, Japan), and operon rubber fiber (polyurethane rubber fiber, KYOUJUDO, Kanagawa, Japan) were used. Measurements with a digital micrometer approximated the ring band as a rectangle with a square cross-section of 1.10 mm × 1.10 mm and the operon rubber fiber as a cylinder with a diameter of 0.40 mm. The following composite materials were prepared in a borosilicate glass screw-tube bottle (20 mL volume, AS ONE Corporation, Osaka, Japan) with a polyethylene packing sheet inside a polypropylene cap.

To incorporate LMWGs, namely alkylhydrazides, into polymeric materials, rubber polymeric materials (rubber rings and operon rubber fibers) underwent initial pretreatment for cleaning. Rubber polymers, cut to lengths of either 3 cm for rubber bands or 5 cm for the operon rubber, were heated in toluene for 30 min and vacuum-dried at 30 °C for 48 h. Subsequently, the pretreated rubber polymer materials were immersed in their respective 3 wt.% LMWG toluene mixture. Heating at 110 °C for 15 min dissolved the LMWGs in toluene, swelling the polymer material with LMWG toluene solution and compositing the LMWGs into the polymer material. Upon cooling the polymeric material/LMWG toluene solution to room temperature, LMWG/polymer composite materials were obtained within a LMWG toluene gel. The resulting LMWG composite polymer material was removed from the gel and vacuum-dried at 30 °C for 48 h. 

To improve the properties of operon rubber containing LMWGs by heat treatment, the heat treatment temperature was set at 45 °C because the endothermic temperature of alkylhydrazides is 40–50 °C. After heating in an oven for 24 or 72 h, changes in structure were evaluated by small-angle X-ray scattering measurements and changes in mechanical properties were evaluated by tensile tests. The changes in structure were evaluated by small-angle X-ray scattering, and the changes in mechanical properties were evaluated by tensile testing.

Thermal analysis by DSC measurement was performed using a high-sensitivity differential scanning calorimeter: DSC7000 (Hitachi High-Tech Corporation, Tokyo, Japan). The temperature was increased and decreased at a rate of 10 °C/min under a nitrogen atmosphere of 200 mL/min from 30 °C to 130 °C or 140 °C to −10 °C, and the heat input to and output from the sample was observed. The transition temperature from gel to sol or sol to gel was defined as the onset temperature between the baseline of the obtained DSC curve and the curve after the change.

SAXS measurements were performed using an automated multipurpose X-ray diffractometer (XRD) with guidance software, Rigaku SmartLab 9 kW (Rigaku Corporation, Tokyo, Japan). CuKα rays (1.54 Å) were used as the X-ray source with copper as the target, and X-rays transmitted through the LMWG composite polymer samples were observed at 25 °C.

Polarized light microscopy was performed using a Leica ML9300 polarizing optical microscope (Meiji Techno Co., Ltd., Saitama, Japan) equipped with a Microscope Camera SwiftCam SC503-CK (Swift Optical Instruments, Inc., Schertz, TX, USA) under crossed Nicols conditions (Saitama, Japan), and the LMWG composite polymer sample was placed on a glass slide.

Scanning electron microscopy measurements were performed using a JSM-6700FN scanning electron microscope (JEOL Ltd., Tokyo, Japan) at 1.0 keV. The LMWG composite polymer samples were carefully placed on a conductive tape on a brass SEM stage with the sample cross-section facing up and were measured after a conductive treatment in which a gold layer was applied to the sample at a thickness of 10 nm using a magnetron sputtering coater.

Infrared absorption was measured by ATR-FTIR using an FTIR6600 spectrometer (JASCO Corporation, Tokyo, Japan) and a single-bounce diamond attenuated total reflectance unit. The resolution was 4 cm^−1^ at 25 °C with 16 integrations.

Tensile tests were performed using a tabletop universal testing machine: MCT-2150 (A&D Company, Limited, Tokyo, Japan). The measurement conditions for the tensile testing of samples were as follows: sample length between fixtures: 10 mm; sample cross-sections: 1.2 mm^2^ (rubber bands) and 0.5 mm^2^ (operon rubber); and test speed: 200 mm/min.

## Figures and Tables

**Table 1:** Weight values of LMWGs compositing into polymeric materials.

LMWG Samples	Increase in Weight after Drying for Ring Band (RB)/%	Increase in Weight after Drying for Operon Rubber (PU)/%
C8HD/C16HD/C18HD:10/1/1	19.0	16.2
C8HD/C16HD/C18HD:1/1/1	14.6	15.7
C8HD	12.4	16.6
C16HD	18.4	14.4
C18HD	10.6	17.9

**Table 2:** Transition temperatures and ΔH of the RB and PU samples obtained through differential scanning calorimetry (DSC) measurements (heating and cooling rate is 10 °C/min).

Samples ^1^	T_melt_ on Heating/°C (ΔH/mJ mg^−1^)	T_crystallization_ on Cooling/°C (ΔH/mJ mg^−1^)
RB-C8HD/C16HD/C18HD 10/1/1	71.4 (18.3)	72.2 (18.0)
RB-C8HD/C16HD/C18HD 1/1/1	74.3 (13.4)	87.4 (13.3)
RB-C8HD	78.4 (16.2)	74.2 (16.3)
RB-C16HD	111.2 (35.1)	106.2 (34.2)
RB-C18HD	10.9 (28.5)	105.2 (28.3)
PU-C8HD/C16HD/C18HD 10/1/1	39.5 (10.9)	43.3 (10.9)
PU-C8HD/C16HD/C18HD 1/1/1	54.8 (5.5)	50.4 (5.8)
PU-C8HD	42.2 (6.1)	32.2 (6.4)
PU-C16HD	94.4 (17.0)	84.3 (15.9)
PU-C18HD	81.3 (12.3)	84.5 (12.4)

^1^ Transition temperatures are defined as the onset temperature of the peaks of DSC curves.

**Table 3:** Mechanical properties obtained from tensile test results of the RB and PU samples.

Samples	Young’s Modulus/MPa	Tensile Strength/MPa (Elongation at Yield/%)
RB	9.8 × 10^−1^	6.4 (258)
RB-C8HD/C16HD/C18HD 10/1/1	1.0	9.5 (332)
RB-C8HD/C16HD/C18HD 1/1/1	1.0	8.8 (335)
RB-C8HD	1.3	9.8 (305)
RB-C16HD	1.1	1.0 × 10 (253)
RB-C18HD	1.8	1.1 × 10 (305)
PU	5.2	2.5 × 10 (380)
PU-C8HD/C16HD/C18HD 10/1/1	4.6	2.5 × 10 (464)
PU-C8HD/C16HD/C18HD 1/1/1	3.7	2.7 × 10 (501)
PU-C8HD	4.3	2.8 × 10 (497)
PU-C16HD	4.0	2.8 × 10 (479)
PU-C18HD	3.6	2.5 × 10 (451)

**Table 4:** Mechanical properties obtained from tensile test results after heat treatment.

Samples ^1^	Young’s Modulus/MPa	Tensile Strength/MPa (Elongation at Yield/%)
PU (before heat treatment)	5.2	2.5 × 10 (380)
PU 1d	6.3	2.5 × 10 (347)
PU 3d	6.2	2.5 × 10 (361)
PU-C8HD/C16HD/C18HD 1/1/1 1d	5.0	2.9 × 10 (503)
PU-C8HD/C16HD/C18HD 1/1/1 3d	5.2	2.7 × 10 (514)
PU-C8HD 1d	5.0	3.2 × 10 (521)
PU-C8HD 3d	5.0	2.9 × 10 (447)

^1^ “d” represents “day”.

## Data Availability

The data presented in this study are available on request from the corresponding author.
